# Steroid treatment in the management of destructive thyrotoxicosis induced by PD1 blockade

**DOI:** 10.1530/ETJ-22-0030

**Published:** 2022-05-27

**Authors:** Alessandro Brancatella, Laura Pierotti, Nicola Viola, Isabella Lupi, Lucia Montanelli, Chiara Cremolini, Paolo Piaggi, Antonio Chella, Andrea Antonuzzo, Daniele Sgrò, Lucia Antonangeli, Chiara Sardella, Sandra Brogioni, Claudio Marcocci, Ferruccio Santini, Francesco Latrofa

**Affiliations:** 1 Endocrinology Unit, Department of Clinical and Experimental Medicine, University Hospital of Pisa, Pisa, Italy; 2 Oncology Unit, Department of Translational Research and New Technologies in Medicine and Surgery, University of Pisa, Pisa, Italy; 3 Department of Information Engineering, University of Pisa, Pisa, Italy; 4 Pneumology Unit, Azienda Ospedaliero-Universitaria Pisana, Pisa, Italy

**Keywords:** immunotherapy, immune check point inhibitors, immune-related adverse event, thyroid, thyroid dysfunction

## Abstract

**Objective:**

Destructive thyroiditis is the most common endocrine immune-related adverse event (iRAEs) in patients treated with anti-PD1/PD-L1 agents. Given its self-limited course, current guidelines recommend no treatment for this iRAE. Nevertheless, in patients with enlarged thyroid volume and a poor performance status, thyrotoxicosis may be particularly severe and harmful. The aim of the study is to evaluate if steroid treatment might be useful in improving thyrotoxicosis in subjects with a poor performance status.

**Methods:**

We conducted a retrospective study, comparing the course of thyrotoxicosis of four patients treated with oral prednisone at the dosage of 25 mg/day (tapered to discontinuation in 3 weeks) and an enlarged thyroid volume to that of eight patients with similar thyroid volume who were left untreated.

**Results:**

The levels of thyroid hormones were lower in subjects treated compared to those untreated at time of 7, 14, 21, 28, 35, 42, 60 and 90 days (*P*  < 0.05 at each time). The time to remission of thyrotoxicosis was 24 days in patients treated with steroids and 120 days in untreated patients (*P*  < 0.001). At 6 months, the rate of evolution to hypothyroidism was similar in the two groups (4/4 in the steroid group vs 7/8 in the untreated group, *P*  = 0.74) and no difference was found in tumor progression (*P*  = 0.89).

**Conclusions:**

Our preliminary data suggest that in patients with a poor performance status experiencing a severe destructive thyrotoxicosis induced by PD-1 blockade, a short period of administration of oral prednisone is effective in obtaining a quick reduction of the levels of thyroid hormones.

## Introduction

In the last few years, the use of immune checkpoint inhibitors (ICIs) in the treatment of several types of solid cancers greatly increased. ICIs are monoclonal antibodies blocking regulatory molecules expressed on T cells, such as cytotoxic T lymphocyte antigen 4 (CTLA-4) and programmed cell death protein-1 (PD1), or non-lymphoid tissues, such as programmed cell death protein ligand 1 (PD-L1) found on tumor cells. By blocking their targets, these antibodies favor the activation of the immune system against cancer and are effective in the treatment of many solid tumors (1, 2). The hyperactivation of the immune system induced by ICIs can also induce several inflammatory tissue reactions known as immune-related adverse events (irAEs) (2). Endocrine glands are frequently involved in irAEs. Thyroid dysfunction and hypophysitis (frequently as isolated adrenocorticotrophic hormone deficiency) are the most common endocrine irAEs, followed by type 1 diabetes and adrenalitis, whereas autoimmune hypoparathyroidism and diabetes insipidus have been reported in isolated cases (3, 4, 5).

We recently characterized two different types of thyrotoxicosis induced by PD1/PD-L1 blockade, a type 1 characterized by persistent hyperthyroidism responsive to anti-thyroid drugs and a type 2, more common, characterized by a painless destructive thyroiditis that usually has a self-limited course (6). In addition, we have shown an association between enlarged thyroid volume and severity and duration of thyrotoxicosis (6). It must be highlighted that in patients with a poorer performance status thyrotoxicosis has been linked to a worst prognosis (7, 8). Although steroid treatment is commonly used in different types of destructive thyroiditis (i.e. subacute thyroiditis and type 2 amiodarone-induced thyrotoxicosis) (9, 10), recent guidelines and expert opinion recommend no treatment for the destructive forms induced by immunotherapy, since data in this setting are lacking (3, 11). Moreover, there are concerns about the possible reduction of the efficacy of immunotherapy in patients treated with steroids (12). Nevertheless, steroids are commonly used for the management of life-threatening iRAEs, namely dermatitis, pneumonia and colitis (13).

In this study, we evaluated the efficacy and safety of steroid treatment in patients with a poor performance status (Eastern Cooperative Oncology Group (ECOG)) performance status score >2) experiencing thyrotoxicosis induced by ICIs.

## Materials and methods

### Study design and population

This was a small retrospective study assessing the utility of glucocorticoid administration in correcting thyrotoxicosis that develops upon PD1/PDL1 blockade. From January to June 2021, 34 patients were referred from Oncology and Pneumology units to the Endocrinology unit of University Hospital of Pisa because of destructive thyrotoxicosis due to anti-PD1 and anti-PD-L1 treatment for metastatic solid cancers. Destructive thyrotoxicosis was defined as the finding of high levels of free thyroxine (FT4) and free triiodothyronine (FT3) associated with low to undetectable levels of thyrotropin (TSH) and absent uptake at ^99m^Technecium (Tc) scintiscan (6). Of these, 4 subjects were treated with oral prednisone at the dosage of 25 mg/day (tapered to discontinuation in 3 weeks) because of a poor performance status (ECOG score >2) (14) and an enlarged thyroid volume. The decision to treat patients was based upon the individual judgment of the endocrinologist, in agreement with an oncologist, any time the severity of thyrotoxicosis was considered a hazard for the patient, in the absence of contraindications to steroid treatment. The starting dosage of steroids was empirically established, based on the typical dosage used in other forms of destructive thyroiditis, namely subacute thyroiditis.

We compared the course of thyrotoxicosis of these subjects to that of eight patients who experienced destructive thyrotoxicosis in the same period who matched thyroid volume at the onset. The study was conducted in accordance with the ethical principles of the Declaration of Helsinki. Data publication was approved by the local institutional review committee (Comitato Etico di Area Vasta Nord Ovest – CEAVNO). Patients were informed and gave their consent to participate in the study.

### Outcome measures

The primary aim of the study was to compare the course of thyrotoxicosis and related symptoms in patients treated with steroids with those observed in untreated subjects. Clinical data (heart rate, blood pressure and thyrotoxicosis-related symptoms) were investigated at the onset of thyrotoxicosis and were collected at time of 30, 90, 150 and 180 days. Thyroid function (FT4, FT3 and TSH) was tested at screening, before the start of immunotherapy (baseline) and at each drug infusion. After the onset of thyrotoxicosis (day 0), thyroid function was tested every 7–10 days in both groups in the first 2 months and then monthly. The minimum follow-up was 6 months in all patients (median 9.2 months). Thyroglobulin antibodies (TgAbs), thyroperoxidase antibodies (TPOAbs) and urinary iodine were tested at screening and at day 0 in all patients, whereas thyroglobulin (Tg) was tested at day 0 and every 28 days thereafter. TSH receptor antibodies (TRAbs) were measured at time 0 in all patients. Neck ultrasound and thyroid scintigraphy were performed at the onset of thyrotoxicosis.

Secondary aims of the study were to evaluate (i) the effect of steroids on the rate of the evolution of thyrotoxicosis to hypothyroidism; (ii) the effect of steroids on antitumoral efficacy of immunotherapy; (iii) the effect of steroid treatment on blood pressure and glucose metabolism.

Hypothyroidism was diagnosed on the basis of two consecutive findings of low FT4 associated with slightly increased TSH (4–10 mIU/L) or of a single detection of low levels of FT4 associated with high TSH (>10 mIU/L).

The response of the oncological disease was evaluated at 6–8 months from the onset of thyrotoxicosis in both groups, according to the response evaluation criteria in solid tumors (15).

Fasting glucose and glycosylated hemoglobin were measured at the onset of thyrotoxicosis in all patients. The same parameters were also measured at the end of steroid treatment in the steroid group and after 150–180 days in untreated patients.

### Laboratory exams

Thyroid hormones and TSH were tested using immunoenzymatic assays (Ortho-clinical diagnostic Inc., Rochester, NY, USA). Reference ranges were 8–18 ng/dL for FT4, 2.5–5.0 ng/L for FT3 and 0.4–4 mIU/L for TSH. Tg was measured by an immunometric assay (Access Thyroglobulin assay; Beckman Coulter, Inc., Fullerton, CA, USA) (functional sensitivity 0.1 ng/mL). TgAbs were measured by AIA-Pack 2000 TgAb-IgGs (Tosoh Corporation, Tokyo, Japan); analytic, functional and positivity cut-offs were 6, 8 and 30 IU/mL, respectively. In this assay, TgAbs interfere with Tg measurement when ≥9.3 IU/mL (16). TPOAbs were checked by AIA-Pack 2000 TPOAb (Tosoh Corporation) (positivity cutoff >10 IU/mL). TRAbs were tested by ELISA (ElisaRSR^TM^ TRAb 3rd generation, Cardiff, UK) (positivity cutoff > 1.5 IU/mL). Urinary iodine was measured by mass-spectroscopy (reference range 100–300 μg/L). Fasting glucose and glycosylated hemoglobin were measured using standard methods (reference range 60–100 mg/dL for glucose and 20–38 mmol/mol for glycosylated hemoglobin, respectively).

### Thyroid imaging

Neck ultrasound was performed by Technos (Esaote Biomedica, Genova, Italy) with a 7.5-MHz linear transducer. Thyroid volume was calculated using the ellipsoid volume formula. The upper limit of normal thyroid volume estimated in the reference Italian adult population is 12.1 mL in women and 16.5 mL in men (17).

^99m^Tc scintiscan was performed using a dedicated gamma camera with a parallel-hole collimator, 20 min after the intravenous administration of 3–5 mCi (111–185 MBq) of ^99m^Tc-pertechnetate.

### Statistical analysis

Statistical data analysis was performed using SPSS 21 (IBM Corp.). Data are presented as mean ± s.d. or median with interquartile range, as indicated. The Shapiro–Wilk test was used to assess normality of data distribution of continuous variables. Statistical tests used to compare groups included Student’s *t*-test for normally distributed variables and Mann–Whitney *U* tests for variables with skewed distribution. The chi-squared test or Fisher’s exact test were used to compare counts and frequencies between groups for categorical variables, as appropriate. Pearson’s (R) and Spearman’s (ρ) correlation coefficients were used to quantify association for Gaussian and skewed continuous variables, respectively. Repeated-measures mixed model analysis was used to assess inter-group differences in thyroid hormone concentrations (dependent variables) over time. Specifically, group, time and their interaction (group × time) were considered as fixed effects, while repeated measurements were modeled using a first-order autoregressive covariance structure. *Post hoc* tests using Fisher’s LSD method were conducted to evaluate difference between groups at each time point. The time to remission, defined as the time from the onset of thyrotoxicosis to the normalization of FT4 levels, was compared between the two groups using the Kaplan–Meier method.

## Results

### General characteristics of study populations

General characteristics of the study populations are summarized in Table 1 and the oncological features of each patient in Table 2.

**Table 1:** General characteristics and thyroid features of the two groups. Weight loss, heart rate, systolic blood pressure, diastolic blood pressure, FT4, FT3, TSH and TgAb are reported as median (25–75th percentile). Number of patients with anxiety, heat intolerance, positive TgAb and TPOAb are also reported.

	Untreated, *n* = 8	Steroid treatment, *n* = 4	*P* value
Clinical data at the onset of thyrotoxicosis			
Mean age (s.d.)	58.3	68.6	0.06
Sex			
Male	5	3	0.98
Female	3	1	
Performance status (ECOG)			
0/1	2	0	0.51
≥2	6	4	
Weight loss from the start of immunotherapy (kg)	3.2 (1.9–4.4)	3.3 (1.8–4.6)	0.38
Heart rate (bpm)	110 (95–120)	112 (100–118)	0.43
Anxiety	4	2	0.99
Heat intolerance	4	2	0.99
Laboratory and imaging features			
FT4 (8–18 ng/dL)	45.5 (49.5–47.2)	48.5 (47.3–49.5)	0.12
FT3 (2.5–5 ng/L)	15.5 (14.5–16.5)	15.0 (12.7–17.2)	0.7
TSH (0.4–4 mIU/L)	0 (0.01–0.1)	0 (0–0)	0.8
TgAb (<30 IU/mL)			
Positive	1	2	0.23
Level	180	150 (32.2–335.5)	
TPOAb (<10 IU/mL)			
Positive	0	0	
Level			
TRAb (<1.5 IU/mL)			
Positive	0	0	
Urinary iodine (100–300 μg/L)	214.2 (180–278)	218.5 (179–287)	0.11
Thyroglobulin (<30 ng/mL)	60.3 (11–480)	51 (6–550)	0.07
Thyroid volume (6–16 mL)	33 (26.7–38.5)	32 (27–38)	0.97
Clinical data at end of follow-up^a^			
Performance status (ECOG)			
0/1	2	0	0.51
≥2	6	4	
Systolic blood pressure (mmHg)	128 (119–148)	124 (108–139)	0.18
Diastolic blood pressure (mmHg)	71 (65–83)	70 (67–88)	0.43
Fasting glucose (mg/L)	105 (85–112)	103 (80–118)	0.68
Glycosylated hemoglobin (mmol/mol)	44 (40–54)	43 (40–56)	0.74

^a^Evaluated at the end of steroid treatment in the treated group and at the end of follow-up in untreated subjects.

FT4, free thyroxine; FT3, free triiodothyronine; Tg, thyroglobulin; TSH, thyrotrophin; TgAb, thyroglobulin antibodies; TRAb, TSH receptor antibodies; TPOAb, thyroperoxidase antibodies.

**Table 2:** Oncological features of the study groups.

Group	Sex	Age	Type of cancer	Stage^a^	Drug	Line treatment	Time from ICI start and thyrotoxicosis onset – days	Best response	PFS from ICI start - months	PFS from thyrotoxicosis onset – months
Untreated	M	56	NSCLC	III C	Pembro	2nd	20	PR	6.0	4.6
Untreated	M	58	Melanoma	IV	Nivo	1st	60	SD	9.0	7.0
Untreated	M	60	NSCLC	IV A	Pembro	3rd	40	PR	12.0	10.6
Untreated	M	54	Melanoma	IV	Nivo	1st	20	SD	9.0+	8.3+
Untreated	M	63	NSCLC	IV A	Nivo	3rd	40	PR	3.0+	1.7+
Untreated	F	72	NSCLC	IV A	Pembro	2nd	60	SD	6.0+	4.0+
Untreated	F	59	Kidney cancer	III C	Pembro	2nd	40	SD	9.0+	5.6+
Untreated	F	45	Kidney cancer	IV B	Pembro	2nd	40	SD	6.0	4.6
Steroid	M	73	Esophagus cancer	IV	Pembro	2nd	30	PR	6.0	5.0
Steroid	M	66	NSCLC	IV A	Nivo	3rd	60	PR	3.0	1.0
Steroid	M	58	NSCLC	IV B	Nivo	2nd	15	SD	9.0	8.5
Steroid	F	78	NSCLC	IV B	Pembro	2nd	30	SD	6.0+	5.0+

^a^According to the American Joint Committee on Cancer tumor, node, metastasis staging (TNM) 8th and 9th editions.

F, female; ICI, immune check point inhibitor; M, male; Nivo, nivolumab; NSCLC, non-small cells lung cancer; PR, partial response; PFS, progression free survival; Pembro, pembrolizumab; SD, stable disease; +, no evidence of progression of disease at last evaluation.

Before the start of immunotherapy (baseline), all subjects had normal thyroid function (FT4 12.8 ng/dL, FT3 3.1 ng/L and TSH 2.0 mIU/L) and negative TgAbs and TPOAbs. Mean age, sex distribution, type of tumors and type of immunotherapy drugs were similar in the two groups (Tables 1 and 2). At the onset of thyrotoxicosis, all patients in both groups experienced tachycardia, with a superimposable heart rate. No difference was observed between the two groups in the other thyrotoxicosis-related symptoms, namely weight loss, blood pressure and anxiety. Similarly, the levels of thyroid hormones, the number of patients with positive thyroid antibodies, the levels of Tg and urinary iodine at the onset of thyrotoxicosis were comparable. The thyroid volume (matching criteria) was enlarged in both groups (Table 1).

### Course of thyrotoxicosis

**Figure 1:**
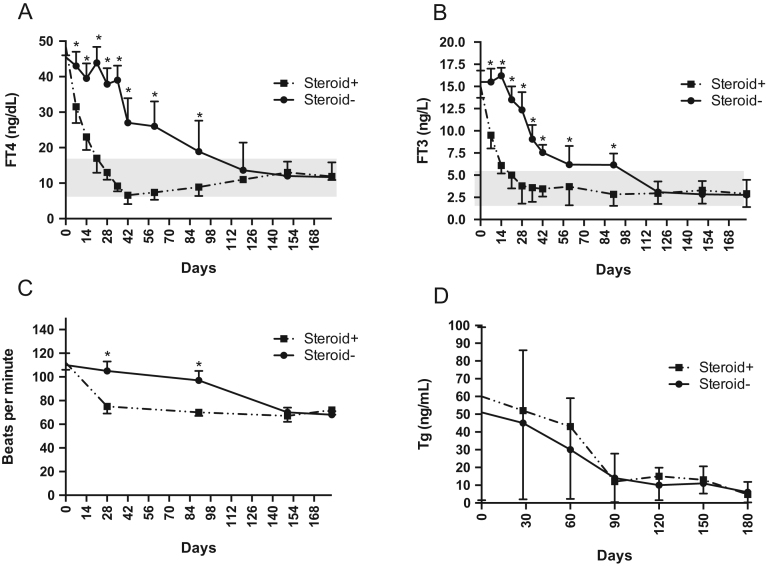
Changes in FT4 (A), FT3 (B), heart rate (C) and thyroglobulin (D) in patients treated with steroids and in untreated patients. Time 0 indicates the onset of thyrotoxicosis. *P  < 0.05 between the two groups.

**Figure 2:**
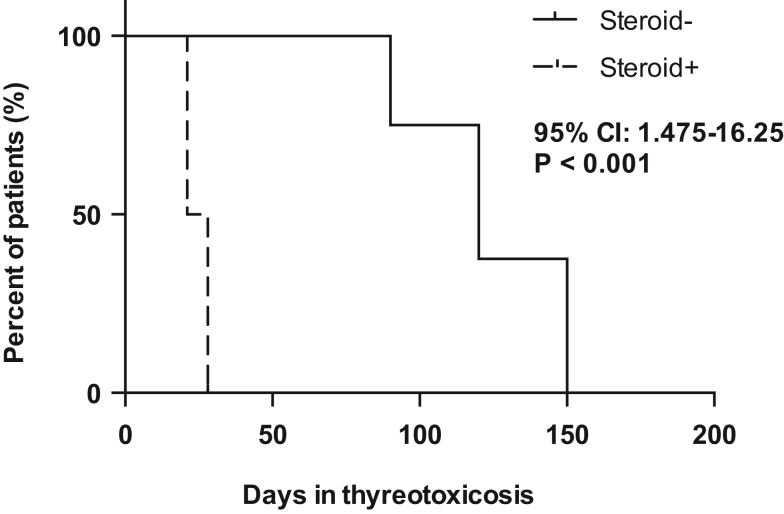
Kaplan-Maier curve showing the time to remission of thyrotoxicosis in the two groups.

The time courses of FT4 and FT3 concentrations over time were different between groups (both group×time interaction *P*  < 0.001), such that the rates of decrease in FT4 and FT3 concentrations were greater in subjects treated with prednisone (β = −0.26 ng/dL/day and β = −0.10 ng/L, respectively) compared to untreated patients (β = −0.15 ng/dL/day and β = −0.05 ng/L, respectively). Specifically, the levels of FT4 and FT3 were lower in subjects treated with prednisone compared to those untreated at time of 7, 14, 21, 28, 35, 42, 60 and 90 days (*P*  < 0.05 at any time). After this time, no difference was found between the two groups (Fig. 1, Panels A and B). The median time to remission of thyrotoxicosis was 24.5 days in patients treated with steroid and 120 days in untreated patients (95% CI 1.47–16.25, *P*  < 0.001) (Fig. 2).

Heart rate was lower in subjects treated with prednisone compared to those untreated at time of 28 and 90 days (*P*  < 0.05 at any time) (Fig. 1, Panel C). At the end of follow-up, thyrotoxicosis-related symptoms disappeared in all patients.

The levels of Tg measured in subjects with negative TgAbs decreased similarly in the two groups across follow-up (*P*  >  0.05 at any time) (Fig. 1, Panel D).

### Onset of hypothyroidism

At 90 days from the onset of thyrotoxicosis, all subjects treated with prednisone and one untreated subject were hypothyroid (*P*  = 0.002). They were then treated with levothyroxine (median dosage of 1.4 mg/kg). After 180 days, no difference was found in the rate of hypothyroidism between the two groups (4/4 vs 7/8, *P*  = 0.74).

### Tumor response

After the onset of thyrotoxicosis, three of four subjects treated with prednisone and two of eight untreated subjects withdrew immunotherapy. All but one started again immunotherapy 21 days (range 14–40 days) after discontinuation. At the end of follow-up, the progression free survival was similar in the two groups (5.0 months in untreated patients vs 5.1 months in steroid group, *P*  = 0.89) (Table 2).

### Safety of steroid treatment

No adverse events were reported by patients treated with steroids. Blood pressure, fasting glucose and glycosylated hemoglobin measured at the end of steroid treatment were similar to those measured in untreated patients at the end of follow-up (Table 1).

## Discussion

Thyrotoxicosis is the most common endocrine iRAE in patients treated with anti PD-1/PD-L1 antibodies, affecting up to 20–40% of subjects (3). Both hyperthyroidism and destructive thyroiditis have been reported during therapy with PD-1/PD-L1 drugs (3, 18). In a recent study, we described two different types of thyrotoxicosis, a type 1 (Graves’ disease like), characterized by persistent hyperthyroidism, and a type 2 (painless destructive thyroiditis) which is more common and usually self-limited (6). Thyroid scintigraphy at the onset of thyrotoxicosis is the most accurate tool to differentiate the two types of thyrotoxicosis and only type 1 thyrotoxicosis is responsive to anti-thyroid drugs (6). Recent guidelines and expert opinion recommend no treatment for the destructive form induced by immunotherapy because of its self-limited course (3, 11). Nevertheless, we observed that destructive thyrotoxicosis occurring in patients with enlarged thyroid volume may be particularly severe and lasts several weeks (6). In addition, different studies have demonstrated that high levels of circulating thyroid hormones are associated with a higher risk of morbidity and mortality, especially in subjects with a poor performance status (19, 20). Thus, in this study, we wanted to evaluate if steroid treatment might be useful in subjects with PD-1/PD-L1 destructive thyrotoxicosis and a poor performance status. For this purpose, we compared the course of thyrotoxicosis in 4 patients with destructive thyrotoxicosis treated with a starting dose of 25 mg of oral prednisone to that of 8 patients left untreated, who were matched for thyroid volume. Indeed, we observed that thyroid volume is the only factor influencing the severity and duration of destructive thyrotoxicosis (6). Both the demographic and oncological characteristics and the levels of thyroid hormones at the onset were similar in the two groups. Destructive thyroiditis was painless in all subjects, as previously reported. Interestingly enough, all patients experienced tachycardia at the onset of thyrotoxicosis whereas other typical symptoms, namely anxiety and hot intolerance, were present only in a minority of patients, presumably due to the short interval between the onset of thyrotoxicosis and the clinical evaluation. We observed that the levels of thyroid hormones dramatically decreased in patients treated with prednisone compared to those untreated. Similarly, heart rate decreased faster in treated patients. The rate of evolution to hypothyroidism after 6 months from the onset of thyrotoxicosis was similar to that previously reported and not different in the two groups (3, 18). The levels of Tg at the onset of destructive thyrotoxicosis were relatively low, with a wide variability across the groups. Moreover, the decrease in Tg during the follow-up was similar in the two groups. This finding raises the possibility that some of these patients might have serum TgAbs of M class, which interfere with Tg measurement and are not detected by commercial assays (21). Finally, no difference in clinical features and glucose levels was found between the two groups at the end of follow-up.

Steroids are a common treatment of destructive thyroiditis, namely subacute thyroiditis and amiodarone-induced thyrotoxicosis type 2. The efficacy of steroids on the control of thyrotoxicosis is variable. Indeed, in subacute thyroiditis, steroid treatment is useful in improving neck pain and general symptoms, whereas the efficacy in the reduction of thyroid hormones is inconsistent (9, 22, 23). Conversely, different studies have demonstrated that steroids have a key role in restoring euthyroidism in patients with type 2 amiodarone-induced thyrotoxicosis (10). Data on the use of steroids in the management of immunotherapy-induced thyroiditis are lacking and conflicting. In their study, Morgenstain and colleagues observed incidentally that patients treated with steroids for other immune-related adverse events had a lower risk of developing thyroid dysfunction (24). Conversely, in a retrospective study by Chanjuan evaluating a group of patients with thyrotoxicosis treated with a high dose of steroids, treatment did not impact the duration of thyrotoxicosis and the rate of evolution to hypothyroidism (25). However, the absence of a stratification of the patients on thyroid volume makes their results of difficult interpretation. Indeed, in the majority of patients, destructive thyrotoxicosis is short and self-limited, given the normal thyroid volume (6). For this reason, we suggest steroid treatment when thyrotoxicosis is associated with an enlarged thyroid volume and therefore is likely to be severe and lasting several weeks.

Some concerns on the use of steroids in the treatment of destructive thyroiditis could derive from the potential reduction of anti-cancer efficacy of immunotherapy (12). Indeed, the data sheet of anti PD1/PD-L1 drugs does not recommend the administration of prednisone at a dosage higher than 10 mg daily. For this reason, we decided to temporally withdraw the immunotherapy in three of four patients treated with steroids. Nevertheless, our data demonstrated that a very short period of steroid treatment is sufficient to induce a quick remission of thyrotoxicosis, enabling a prompt restart of immunotherapy. In addition, in accordance with recent studies that have shown that a dosage higher than 10 mg of prednisone during immunotherapy is not associated with a worse prognosis, we observed no difference in tumor response between the two groups (26). It is worth noting that severe thyrotoxicosis may indicate that immunotherapy should be interrupted (27).

Further studies, investigating larger cohorts of patients, are required to identify patients who may benefit from this treatment and to establish the duration and the appropriate dosage of steroids treatment. In addition, larger cohorts are also needed to better investigate the safety of steroid treatment and its potential effect on the anti-tumoral efficacy of immunotherapy.

In conclusion, our preliminary data suggest that in patients with a poor performance status experiencing a severe destructive thyrotoxicosis induced by PD-1 blockade, a short period of administration of oral prednisone is effective in obtaining a quick reduction of the levels of thyroid hormones and related symptoms.

## Declaration of interest

The authors declare that there is no conflict of interest that could be perceived as prejudicing the impartiality of the research reported.

## Funding

This work was supported by ‘Fondi di Ateneo’, University of Pisa to Francesco Latrofa.

## Statement of ethics

The study was conducted in accordance with the ethical principles of Declaration of Helsinki. Data publication was approved by the local institutional review committee (Comitato Etico di Area Vasta Nord Ovest – CEAVNO). Patients were informed and gave their consent to participate in the study.

## Data availability statement

Some or all data generated or analyzed during this study are included in this published article or in the data repositories listed in references.

## Author contribution statement

A B, L P, and F L planned the study. A B, L P, C C, and F L wrote the manuscript. P P performed statistical analysis. All authors discussed the results of the study.
